# Hybrid Pt/CeO_2_/TiO_2_ Catalysts for Low-Temperature Iron(II) Ion Oxidation with Oxygen in Acidic Media

**DOI:** 10.3390/nano16191221

**Published:** 2026-09-27

**Authors:** Nikolay S. Ivanov, Sergey K. Oparin, Nataliya A. Ivanova, Oleg S. Kholkin, Iskander E. Adelbayev, Vladislav Kudryashov, Arlan Z. Abilmagzhanov

**Affiliations:** 1 D.V. Sokolsky Institute of Fuel, Catalysis and Electrochemistry JSC, 142 Kunayev Str., Almaty 050010, Kazakhstan; xumuk777@mail.ru (N.S.I.); a.abilmagzhanov@gmail.com (A.Z.A.); 2 Al-Farabi Kazakh National University, 71, Al-Farabi Avenue, Almaty 050012, Kazakhstan; 3 Renewable Energy Lab, “National Laboratory Astana”, Nazarbayev University, 53 Kabanbay Batyr Ave., Astana 010000, Kazakhstan

**Keywords:** magnesium leaching, goethite process, iron(II) ion oxidation, mixed oxide support, platinum deposition, Pt/CeO_2_/TiO_2_ catalysts, acidic media, ceria nanoparticles

## Abstract

The presence of iron(II) impurities hinders the leaching and purification of magnesium ores. The most viable technological solution is to oxidise Fe^2+^ to Fe^3+^, which then precipitates as goethite. However, this process requires the development of new catalysts for use in highly acidic media. This study demonstrates that the Pt^0.25^/CeO_2_^5^/TiO_2_ catalyst is highly effective at oxidising Fe^2+^ in 0.3 M HNO_3_, outperforming samples supported on pure titanium dioxide or cerium dioxide. The rate constant is 0.304 L/(mol·min) at room temperature. Notably, the activation energy is only 10.7 kJ/mol, which is over five times lower than that of the non-catalytic process. This result was achieved by selectively depositing platinum particles onto highly dispersed cerium dioxide particles (average size ~5 nm), synthesised using the sol–gel method, and then depositing them onto titanium dioxide. Using a hybrid mixed oxide support ensures a high density of oxygen vacancies at the interface between the platinum (Pt) and mixed cerium and titanium dioxide phases. The formation of abundant adsorbed oxygen species enhances the catalytic activity of the Pt^0.25^/CeO_2_^5^/TiO_2_ catalyst. The catalyst Pt^0.25^/CeO_2_^5^/TiO_2_ also reaches a plateau in terms of efficiency after a durability experiment, which is the highest among the other samples.

## 1. Introduction

Iron is the fourth most abundant element in Earth’s crust [1,2] and a constituent of numerous minerals [3,4,5]. Its presence leads to iron contamination of solutions during the hydrometallurgical processing of ores [6,7,8], and the selective precipitation of iron remains challenging due to the co-precipitation of target metals. For example, this occurs during magnesium leaching. The three primary methods of removing iron salts from solutions are the Jarosite process, the Goethite process, and the Hematite process. A key advantage of the Goethite process is that it forms large goethite (FeOOH) crystals [9,10], which are easier to filter from the solution. Goethite phase formation proceeds via the oxidation of iron(II) to iron(III) [10,11]. However, the oxidation of iron(II) by atmospheric oxygen is very slow in acidic media [12]. According to [13], the rate-limiting step of this reaction in an acidic medium (pH = 4) at temperatures of 70–90 °C is electron transfer to oxygen. Specifically, the activation energy for the oxidation of Fe^2+^ to Fe^3+^ in 1 M H_2_SO_4_ is 62.1 kJ/mol [14]. The slow kinetics of the process are a particularly critical factor during low-temperature oxidation. Consequently, intensifying iron oxidation in acidic media using catalysts is a current area of interest.

The literature reports the use of several homogeneous catalysts for the Fe^2+^ oxidation process. The authors of [15] investigated the use of Cu^2+^ ions as catalysts for oxygen-mediated Fe^2+^ oxidation in a 1–2 M H_2_SO_4_ medium. The activation energy found for this process was 36.5 kJ/mol, half that observed under similar conditions in the absence of catalysts [14]. Nitrite ions have also been shown to act as homogeneous catalysts for Fe^2+^ oxidation [16,17]. The catalytic oxidation mechanism involves the formation of NO and NO_2_ during the process, which continuously oxidise Fe^2+^ in the presence of oxygen. While nitrites yield excellent results in terms of the rate of Fe^2+^ oxidation, consumed over the course of the reaction over time, increasing pH.

Considering these gaps, researchers have attempted to adapt heterogeneous catalysts for the Fe^2+^ oxidation process. Materials investigated for this purpose include activated carbon [18] (with an activity of *k* = 0.187 M^−0.5^·min^−1^ at 80 °C), ferrihydrite [19] (with an activity of *k* = 2.12 M^−1^·s^−1^ at pH 4.5–5.5 and 25 °C), and platinum on carbon [20]. Platinum is an excellent catalyst due to its ability to lower the energy barrier for oxygen-based oxidation reactions [21,22,23]. However, the supports used for platinum in the previous studies, such as carbon, are susceptible to degradation and oxidation in highly acidic media [24]. Meanwhile, iron oxide is catalytically inactive in acidic environments (pH < 1). Therefore, finding new, stable catalysts and supports for the Fe^2+^ oxidation reaction in highly acidic media remains a pressing challenge.

Titanium and cerium oxides occupy a unique position among platinum catalyst supports due to their stability in acidic environments, a property that is particularly evident in cerium dioxide. Interest in Pt/TiO_2_ and Pt/CeO_2_ systems stems from the ability of both oxides to form oxygen vacancies and reduced centres, which create a distinct metal–support interaction [25] at the interface. This interaction dictates platinum dispersion, electronic state, and surface reactivity [26,27,28,29]. In the case of Pt/TiO_2_, the enhanced interaction is accompanied by an increase in the concentration of Ti^3+^ centres and surface oxygen vacancies. The vacancies themselves participate in O_2_ activation [26,27,28]. For Pt/CeO_2_, it has been established that the oxygen-deficient cerium oxide surface stabilises highly dispersed platinum states by forming a Pt–O–Ce interface [29]. Concurrently, the capacity for oxygen adsorption increases, while the enhanced Pt–CeO_2_ interaction inhibits the migration and agglomeration of platinum particles [26,27]. Therefore, TiO_2_ and CeO_2_ are not only of interest as chemically stable supports but also as defective oxide matrices that can specifically modulate platinum dispersion, electronic state, and oxidative activity [30,31]. This makes them promising supports for catalysts designed to oxidise iron(II) using atmospheric oxygen in acidic media.

Using hybrid supports, in which a finely dispersed cerium dioxide sol is deposited onto titanium dioxide powder, offers an effective way to synthesise this type of catalyst. This approach increases the support’s specific surface area, ensures a uniform distribution of platinum particles, and enhances their durability in acidic media due to the preferential interaction between the platinum particles and the CeO_2_. In the present study, this catalyst composition was achieved using a synthesis method involving the selective deposition of platinum particles onto cerium dioxide. To demonstrate the advantages of a hybrid mixed oxide support, the obtained catalyst samples underwent structural analysis and kinetic testing in the iron(II) ion oxidation reaction in 0.3 M HNO_3_, and the results were compared with those of samples supported on pure titanium dioxide and cerium dioxide phases.

## 2. Materials and Methods

### 2.1. Chemicals and Materials

Titanium dioxide (99%, Sigma-Aldrich, Canada), cerium nitrate (99.5%), hexachloroplatinic acid (99.5%), sodium borohydride (99.8%, Sigma-Aldrich, Darmstadt, Germany), aqueous hydrogen peroxide solution (36% RUSCHIM LLC Moscow, Russia), aqueous ammonia solution (25% RUSCHIM LLC Moscow, Russia), and oxygen (Analytical grade 6.0, Linde Gas, Germany) were used.

### 2.2. Preparation of Catalysts

To investigate the properties of mixed catalysts, TiO_2_-based catalysts in the anatase phase—Pt^0.5^/TiO_2_ and Pt^0.25^/TiO_2_—were prepared, with nominal platinum loadings of 0.5 and 0.25 mass%, respectively. In addition, catalysts Pt^0.5^/CeO_2_^10^/TiO_2_ and Pt^0.25^/CeO_2_^5^/TiO_2_ were synthesized, with nominal Pt contents of 0.5 and 0.25 mass% and an incorporated CeO_2_ nanoparticle content of 10 and 5 mass%, respectively. A Pt^0.5^/CeO_2_ catalyst with a nominal platinum content of 0.5 mass % was prepared as a reference sample. 

#### 2.2.1. Preparation of Pt/TiO_2_ and Pt/CeO_2_ Catalysts

The Pt/TiO_2_ catalyst was prepared using the sorption deposition of platinum from an aqueous solution of H_2_PtCl_6_. First, a TiO_2_ sample was sonicated in distilled water for 90 min. Then, a calculated volume of the aqueous H_2_PtCl_6_ precursor solution corresponding to the target platinum content of the final catalyst (0.5% and 0.25% by weight) was added to the resulting suspension. The suspension was then stirred for 24 h at 80 °C. Once the sorption process was complete, the platinum was reduced using a 5% NaBH_4_ solution. The Pt^0.5^/CeO_2_ catalyst was prepared using a similar sorption method.

#### 2.2.2. Preparation of Pt^0.5^/CeO_2_^10^/TiO_2_ and Pt^0.25^/CeO_2_^5^/TiO_2_ Catalysts

To prepare the Pt^0.5^/CeO_2_^10^/TiO_2_ and Pt^0.25^/CeO_2_^5^/TiO_2_ catalysts, a CeO_2_ sol with varying concentrations of CeO_2_ was first synthesised. First, hydrogen peroxide was added to an aqueous Ce(NO_3_)_3_ solution at a concentration of 0.02 mol/L for Pt^0.5^/CeO_2_^10^/TiO_2_ and 0.01 mol/L for Pt^0.25^/CeO_2_^5^/TiO_2_, with a Ce^3+^:H_2_O_2_ molar ratio of 1:3. The resulting reaction mixture was stirred on a magnetic stirrer at 60 °C for 90 min. An aqueous ammonia solution was then added to adjust the pH to 9.20, and stirring continued at 60 °C for a further 90 min. The obtained CeO_2_ sol exhibited a characteristic orange-yellow colour. Next, a weighed amount of TiO_2_ powder was introduced into the CeO_2_ sol, and the resulting suspension was stirred for 120 min. The resulting hybrid mixed oxide support was then filtered, dried, and calcinated at 450 °C for four hours. For catalyst preparation, an aqueous suspension was prepared from the resulting powder, and an H_2_PtCl_6_ solution was added to achieve a platinum content of 0.5 wt.% for Pt^0.5^/CeO_2_^10^/TiO_2_ and 0.25 wt.% for Pt^0.25^/CeO_2_^5^/TiO_2_, respectively. The reaction mixture was stirred for 24 h, after which a 5% NaBH_4_ solution was added in an amount 20% more than the stoichiometric requirement for complete Pt reduction. The mixture was then stirred at 25 °C for a further 12 h to complete the reduction process.

### 2.3. Structural and Morphological Studies

#### 2.3.1. BET Method

The specific surface area for the synthesised catalyst samples was determined by nitrogen sorption and capillary condensation. This parameter was determined by processing the nitrogen vapor adsorption isotherm at a temperature of 77 K, measured on a volumetric high-vacuum adsorption unit Tristar 3000 (Micromeritics, USA) in the range of relative pressures from 10^−6^ to 0.99 using the volumetric method. The samples were degassed under vacuum at 200 °C for 2 h. The total specific surface area of the supports (*SA_BET_*, m^2^/g) was determined from the adsorption isotherm using the BET method according to the following equation:

(1) SABET = CSA×NaVm×1018 nm2m2×SLP+YINT

where CSA is the cross-sectional area of a nitrogen molecule (0.162 nm^2^), *N_a_* is the Avogadro constant (6.023 × 10^23^ mol^−1^), *V_m_* is the molar volume of gas (22,414 cm^3^), and *S_LP_* and *Y_INT_* are the slope and the vertical axis intercept, respectively, for the linear dependence of *B_i_*_(*pi/pi,0*)_ (g·cm^−3^):

(2) $$B_{i}\;=\;\frac{\frac{p_{i}}{p_{0,i}}}{\left(1\;-\;\frac{p_{i}}{p_{0,i}}\right)\times N_{ads,i}}$$

*p_i_/p_0,i_*, *N_ads,i_*—relative pressure and amount of adsorbed nitrogen (cm^3^·g^−1^) at the *i*-th measurement.

#### 2.3.2. XRD and XRF

X-ray diffraction analysis (XRD): The diffraction patterns of the electrocatalyst powders were obtained using synchrotron radiation (λ = 0.7914 Å) at the Belok/XSA station of the Kurchatov Institute’s synchrotron radiation source (Russia), which is equipped with a Rayonix SX165 2D CCD detector (Evanston, USA). The beam size was 400 × 400 µm^2^, and the distance between the sample and the detector was 150 mm. The exposure time was 5 min. All measurements were carried out at room temperature.

The elemental composition of the samples was determined using a Bruker M4 Tornado spectrometer (Berlin, Germany) equipped with a Rh-anode X-ray tube and Kumakhov polycapillary focusing optics and X-ray fluorescence analysis (XRF). The diameter of the X-ray beam at the sample surface was 25 µm. To reduce X-ray absorption and scattering by air, the analytical chamber was evacuated to a pressure of less than 20 mbar. Fluorescent radiation was detected using two energy-dispersive detectors with 12 µm thick beryllium windows.

#### 2.3.3. TEM

TEM examinations of the samples were carried out using a JEM-2100 transmission electron microscope (JEOL, Japan) equipped with an X-Max EDS detector (Oxford Instruments) at an accelerating voltage of 200 kV. The distributions of the average diameters of platinum and cerium oxide particles were constructed by processing TEM images with ImageJ (National Institutes of Health, USA, version 1.54u8) software.

#### 2.3.4. SEM

A Versa 3D DualBeam scanning electron microscope (FEI, USA) was used to investigate the morphology of the samples. The analysis was conducted under low-vacuum and ESEM (environmental scanning electron microscopy) conditions. An LVSED (low-vacuum secondary electron detector) was used for secondary electron detection in low-vacuum mode, whereas a highly sensitive GSED (gaseous secondary electron detector) was used in environmental mode. For ESEM analysis; during the experiment, the relative humidity of the gas environment in the chamber was reduced from 100% to 20% to reveal additional morphological features of the samples.

#### 2.3.5. XPS

X-ray photoelectron spectroscopy (XPS) was performed using a Thermo Scientific Nexsa XPS system (East Grinstead, UK) with monochromatic Al Kα radiation to determine the chemical states of the prepared catalysts. The catalysts were pre-annealed at 700 °C in an argon atmosphere to remove the surface-blocking layer and increase accessibility to the platinum particles.

### 2.4. Catalytic Testing and Kinetic Experiments

Catalyst testing was conducted in an autoclave-type reactor (ZNCL, TGF-B-500 Zhengzhou Keda Machinery and Instrument Equipment Co., Ltd.) equipped with a magnetic stirrer, thermostat, and temperature control systems. The reactor was connected to an oxygen cylinder via a pressure regulator. Figure 1 shows a schematic of the setup.

Kinetic tests of the catalysts were conducted at an oxygen pressure of 10 atm. to increase the oxygen concentration in the solution while stirring at 600 rpm [32]. The catalyst mass was standardised to a platinum content of 0.75 mg per experiment. The volume of the reaction solution was 100 mL, with an initial Fe^2+^ concentration of 0.10 mol∙L^−1^ and an HNO_3_ concentration of 0.30 mol∙L^−1^ (corresponding to a pH of 0.5). A freshly prepared Fe^2+^ solution was used for each kinetic experiment. The rate constant *k* was calculated using the second-order kinetic Equation (3). The reaction order was determined by graphically differentiating the kinetic curve using the Van’t Hoff method and plotting $\ln\left(-\frac{∆C}{∆t}\right)$ versus $\ln\left(C_{i}\right)$, where the slope (tan φ) corresponds to the reaction order *n*. The Arrhenius plot (Equation (4)) was used to determine the activation energy. Measurements were performed over a temperature range of 20–60 °C, with data collected at a minimum of four points.

(3) $$k=\frac{1}{t}\left(\frac{1}{C\left({\mathrm{Fe}}^{2+}\right)}-\frac{1}{C_{0}\left({\mathrm{Fe}}^{2+}\right)}\right)$$

(4) $$\ln\left(k\right)=f\left(\frac{1}{T}\right)$$

The stability of the catalyst was evaluated in successive 15-min reaction cycles at an oxygen pressure of 10 atm and a stirring speed of 600 rpm. The volume of the Fe^2+^ solution was 50 mL, and a fresh solution was used for each new cycle.

Catalyst activity was assessed by calculating the *turnover frequency* (TOF) using Equation (5).

(5) $$Turnover\;frequency=\frac{n\left({\mathrm{Fe}}^{3+}\right)}{m_{\mathrm{Pt}}\cdot t}$$

The TOF and kinetic characteristics, normalised to the Pt content, were calculated using experimental platinum content values obtained by XRF.

## 3. Results

### 3.1. Characterization of the Catalysts

#### 3.1.1. Composition and Morphology of the Catalysts

The morphology and dispersion of the active Pt phase of the synthesised catalysts were investigated using SEM and TEM (see Figure 2). SEM images of all samples revealed that the catalysts consisted of TiO_2_ particle aggregates ranging from 100 to 300 nm in size. TEM images of the Pt^0.5^/TiO_2_ and Pt^0.25^/TiO_2_ samples reveal platinum particles on the support surface. The Pt^0.5^/TiO_2_ sample exhibits a narrow particle size distribution, with an average diameter of 3–4 nm. Reducing the Pt content to 0.25 wt.% (Pt^0.25^/TiO_2_) results in a broader platinum particle size distribution, with particles in the 8–11 nm range predominating. This effect is attributed to the non-uniform distribution of the precursor on the support surface during adsorption, resulting from the low loading level.

The addition of highly dispersed CeO_2_ particles results in a change in the morphology of the catalyst. SEM and TEM images of the CeO_2_-modified Pt^0.5^/CeO_2_^10^/TiO_2_ and Pt^0.25^/CeO_2_^5^/TiO_2_ samples reveal CeO_2_ nanoparticles that are uniformly distributed across the support surface. For the Pt^0.5^/CeO_2_^10^/TiO_2_ sample, the CeO_2_ particles predominantly have diameters in the range of approximately 5 to 15 nm. For the sample Pt^0.25^/CeO_2_^5^/TiO_2_ prepared with a lower cerium loading, the average CeO_2_ particle size falls within the 4–5 nm range, which is generally consistent with previously reported data [33,34,35]. Both samples exhibit a narrow Pt particle size distribution. For the Pt^0.5^/CeO_2_^10^/TiO_2_ sample, the average Pt particle size is primarily in the 6–7 nm range, whereas for the Pt^0.25^/CeO_2_^5^/TiO_2_ sample, it is in the 4–5 nm range. Thus, a reduction in platinum content is accompanied by a slight shift in the distribution towards smaller sizes. However, increasing the cerium dioxide content leads to a marked increase in agglomeration, resulting in platinum deposition onto the titanium dioxide and an increase in the average Pt particle size. Conversely, lower cerium dioxide content and the formation of smaller CeO_2_ particles facilitate the selective deposition of platinum onto the cerium dioxide.

#### 3.1.2. Structure of the Catalysts

Figure 3 shows the X-ray diffraction patterns of the synthesised catalysts. For the Pt^0.5^/TiO_2_ and Pt^0.25^/TiO_2_ samples, the main set of reflections corresponds to the tetragonal anatase modification (COD No. 1526931), while weak reflections from the rutile phase of TiO_2_ (COD No. 9015662) are also detected as an impurity. In addition to the TiO_2_ reflections, weak reflections corresponding to the CeO_2_ structure (COD No. 1562989) are observed for the Pt^0.5^/CeO_2_^10^/TiO_2_ and Pt^0.25^/CeO_2_^5^/TiO_2_ catalysts. The low intensity of these reflections is consistent with the low CeO_2_ content (5–10 wt.%) in the composite samples. For the Pt^0.5^/CeO_2_ sample, the observed set of intense reflections corresponds entirely to the CeO_2_ phase. Low-intensity characteristic reflections of metallic platinum (COD No. 2104026) are observed in all samples, confirming its presence.

XRF analysis was performed to determine the platinum and cerium content of the catalysts quantitatively. For the Pt^0.5^/TiO_2_ and Pt^0.25^/TiO_2_ samples, the experimentally determined platinum content relative to titanium was 0.80 wt.% and 0.28 wt.%, respectively. For the CeO_2_-modified samples, the platinum content was 0.50 wt.% for Pt^0.5^/CeO_2_/TiO_2_ and 0.15 wt.% for Pt^0.25^/CeO_2_/TiO_2_. For the Pt^0.5^/CeO_2_ sample, the platinum content relative to cerium was 0.70 wt.%. Given the low platinum content, the determination error is approximately 10%. The analysis results are presented in Table 1.

The BET method was used to determine the specific surface area. For Pt^0.5^/TiO_2_ and Pt^0.25^/TiO_2_, the SSA values were 5.78 and 5.20 m^2^·g^−1^, respectively. Modifying TiO_2_ with cerium oxide increased the specific surface area to 7.40 m^2^·g^−1^ for Pt^0.5^/CeO_2_^10^/TiO_2_ and to 9.12 m^2^·g^−1^ for Pt^0.25^/CeO_2_^5^/TiO_2_. This increase correlates well with the addition of cerium oxide nanoparticles. Notably, the highest specific surface area was observed for Pt^0.25^/CeO_2_^5^/TiO_2_, where the nanoparticles exhibited the smallest size and monomodal distribution, according to TEM data. The lowest value, 3.17 m^2^·g^−1^, was obtained for Pt^0.5^/CeO_2_. The obtained data are presented in Table 1.

The O 1s spectra of the Pt/CeO_2_/TiO_2_ and Pt^0.5^/CeO_2_ catalysts (Figure 4b) reveal two notable components with binding energies (BE) of near 529–530 eV and near 531–532 eV, which correspond to lattice oxygen and surface-adsorbed oxygen, respectively [36,37]. The adsorbed oxygen peak is least pronounced for the Pt^0.5^/TiO_2_ catalyst (Table 2). The binding energy of the adsorbed oxygen decreases as the platinum content and the average particle size decreases, which is a result of electron transfer between platinum and oxygen. This is also reflected in the binding energy shift observed in the Pt 4f 5/2 spectra (Figure 4a). The binding energy values for the Pt 4f 7/2 spectra are presented in Figure 4c and show a strong correlation with the average platinum particle sizes in the catalyst samples, as calculated from the obtained TEM images. Binding energy (BE) values for the PtO_x_ 4f 7/2 suggest that the lowest value is for the CeO_2_-based catalyst. This is due to the high concentration of near-surface adsorbed oxygen, resulting from CeO_2_’s ability to form oxygen vacancies [36]. In this case, the major oxidized state of the platinum is due to the interaction of the metal with the support via Pt–O–Ce bonds. Despite its lower CeO_2_ content, the Pt^0.25^/CeO_2_^5^/TiO_2_ sample has a PtO_x_ 4f 7/2 BE value that falls closest to this range, indicating a high degree of CeO_2_ utilisation. However, the adsorbed oxygen content of the catalyst may be underestimated due to sample pretreatment. 

Previous studies have shown that reversible Ti^4+^/Ti^3+^ and Ce^3+^/Ce^4+^ redox cycles in mixed CeO_2_/TiO_2_ oxides lead to the formation of a significantly larger number of oxygen vacancies than in pure CeO_2_ [36]. Furthermore, electron transfer between Pt and Ce species (Pt^0^ + Ce^4+^ and Pt^2+^ + Ce^3+^), particularly with smaller Pt nanoparticles, increases the density of oxygen vacancies at the Pt and mixed CeO_2_/TiO_2_ oxide interface [36]. Previous studies have described this phenomenon based on in situ catalyst analysis. In contrast, the present study examined the initial state of the catalysts. This undoubtedly limits this study. Nevertheless, the similarity of the catalyst compositions and structural analysis results suggests that analogous effects occur in catalysts during the oxidation of iron(II) by oxygen.

Thus, increasing the amount of surface oxygen species has a more pronounced effect on catalysts with lower platinum content and smaller particle sizes. The formation of many adsorbed oxygen species can contribute to the increased catalytic activity of samples in iron(II) ion oxidation reactions, which is a particularly notable feature of the Pt^0.25^/CeO_2_^5^/TiO_2_.

### 3.2. Kinetic Results

To determine the kinetic parameters of the Fe^2+^ oxidation reaction, experiments were carried out using the catalysts under investigation in Figure 5. The order of the reaction was determined using the Van’t Hoff method. For all systems, it was found to be 2. Based on the calculated reaction order, the apparent rate constants were calculated using the integral second-order kinetic equation. The values of the rate constants and activation energies for the catalysts studied are given in Table 3. For the Pt^0.5^/TiO_2_, Pt^0.5^/CeO_2_^10^/TiO_2_, and Pt^0.5^/CeO_2_ catalysts, the rate constants were 0.093, 0.069, and 0.094 L·mol^−1^·min^−1^, respectively. The highest value was obtained for Pt^0.25^/CeO_2_^5^/TiO_2_—0.304 L·mol·min^−1^. For the Pt^0.25^/TiO_2_ catalyst, the reaction order could not be reliably determined because the experimental data fell outside the applicability range of the method used for reaction-order determination. Consequently, in the absence of a reliably established reaction order, the corresponding rate constant could not be determined. The rate constants correlate with the calculated activation energies. The lowest activation energy, 10.7 kJ mol^−1^, was determined for the Pt^0.25^/CeO_2_^5^/TiO_2_ catalyst.

A comparative assessment of the catalytic activity (TOF) of the synthesised Pt-containing catalysts in the oxidation of Fe^2+^ by molecular oxygen was carried out, including an evaluation of stability under repeated cycling (Figure 5d). The TOF values for most of the samples studied were within a similar range and differed only within the experimental margin of error. The exception was the Pt^0.25^/CeO_2_^5^/TiO_2_ catalyst, for which a pronounced TOF maximum was observed during the initial cycles. With further cycling, the activity decreased and reached a quasi-steady-state level, which remained approximately 30 percent higher than the values obtained for Pt^0.25^/TiO_2_, Pt^0.5^/TiO_2_ and Pt^0.5^/CeO_2_. After the fifth cycle, a gradual decrease in TOF was observed for all catalysts; by the 15th cycle, the decrease in activity was approximately 25%.

The catalytic activity of each of the investigated catalysts requires separate consideration. A comparison of the Pt^0.25^/TiO_2_ and Pt^0.5^/TiO_2_ catalysts shows that the higher catalytic activity of Pt^0.5^/TiO_2_ may be attributed to the smaller size of the Pt particles. According to SEM data, Pt^0.5^/TiO_2_ is characterised by particles of approximately 4 nm in size, whereas for Pt^0.25^/TiO_2_ their average size is approximately 10 nm. The higher dispersion of platinum nanoparticles in Pt^0.5^/TiO_2_ ensures a greater proportion of accessible surface platinum centres, which may contribute to an increase in the rate of Fe^2+^ oxidation. A fine-particle size distribution of platinum is also observed for the Pt^0.5^/CeO_2_ catalyst, for which the average Pt particle size is approximately 6 nm; this is close to the particle size in Pt^0.5^/TiO_2_ and is consistent with the similar values of their catalytic activities [38,39,40].

To evaluate the effect of oxygen pressure in the reaction system on the Fe^2+^ oxidation rate, additional experiments were conducted using the Pt^0.25^/CeO_2_^5^/TiO_2_ catalyst at different O_2_ pressures. The kinetic curves for Fe^2+^ oxidation are presented in Figure 6, and the reaction order was also evaluated.

With increasing oxygen pressure, a gradual increase in the reaction rate of Fe^2+^ → Fe^3+^ was observed. The calculated apparent rate constants were 0.1795, 0.2406, and 0.3041 L·mol^−1^·min^−1^ at O_2_ pressures of 2, 6, and 10 atmospheres, respectively. The reaction order with respect to oxygen was also determined from the obtained kinetic data. In all cases, the reaction order was equal to 2 within the experimental uncertainty.

The mechanism of catalytic oxidation of Fe^2+^ by molecular oxygen on the surface of Pt^0.25^/CeO_2_^5^/TiO_2_ warrants special attention, as this sample exhibits the highest catalytic activity amongst the catalysts studied. It is suggested that the enhanced activity of Pt^0.25^/CeO_2_^5^/TiO_2_, compared with Pt^0.5^/TiO_2_ and Pt^0.5^/CeO_2_, is due to the facilitated transfer of reactive oxygen species from the surface of the CeO_2_ nanoparticles to the platinum centres, in the presence of well-developed Pt–CeO_2_ interfacial contact. Oxygen-mediated oxidation processes in the aqueous phase on noble-metal catalysts proceed according to (6)–(8). In these reactions, oxygen dissociates and interacts with adsorbed water molecules to form peroxide species [41,42].

(6) $$O_{2}^{*}+H_{2}O^{*}\to {\mathrm{OOH}}^{*}+{\mathrm{HO}}^{*}$$

(7) $${\mathrm{OOH}}^{*}+H_{2}O^{*}\to \;H_{2}O_{2}^{*}+{\mathrm{HO}}^{*}$$

(8) $${\mathrm{HO}}^{*}+e^{-}↔\;{\mathrm{HO}}^{-}+*$$

where *—the bond between the adsorbed molecule and Pt; $O_{2}^{*}$—adsorbed oxygen; $H_{2}O^{*}$—adsorbed water on Pt.

The Fe^2+^ oxidation mechanism can proceed via reactions (9)–(11), which involve the interaction between Fe^2+^ and adsorbed peroxide oxygen species on the platinum surface.

(9) $${\mathrm{Fe}}^{2+}+{\mathrm{HO}}^{*}\;\to \;{\mathrm{Fe}}^{3+}+{\mathrm{HO}}^{-}$$

(10) $${\mathrm{Fe}}^{2+}+{\mathrm{OOH}}^{*}+H^{+}\;\to \;{\mathrm{Fe}}^{3+}+2{\mathrm{OH}}^{-}$$

(11) $$H^{+}+{\mathrm{HO}}^{-}\;\to H_{2}O$$

However, as we suggest, the addition of CeO_2_ nanoparticles to the TiO_2_ support significantly enhances the catalytic activity of platinum, particularly when the CeO_2_ particle size is below 5 nm [43]. The catalyst Pt^0.25^/CeO_2_^5^/TiO_2_ supported on the mixed oxide support exhibits the highest rate constant (0.304 L·mol^−1^·min^−1^) for the Fe^2+^ oxidation reaction, surpassing samples supported on pure TiO_2_ or CeO_2_. This enhancement is due to the transfer of oxygen from the CeO_2_ surface to the platinum particles. Additional oxygen species are supplied by the CeO_2_ via a transfer process driven by the strong metal–support interaction between Pt and CeO_2_ particles, respectively [39,44]. This effect is particularly pronounced as the size of the CeO_2_ nanoparticles decreases, accompanied by an increased concentration of oxygen vacancies [43,45,46], indicating more efficient oxidation processes. Oxygen vacancy formation can be described by the corresponding quasi-chemical Equations (12) and (13). According to Schottky:

(12) $${\mathrm{Ce}}_{\mathrm{Ce}}^{\times }\;+\;2O_{O}^{\times }\;↔\;V_{\mathrm{Ce}}^{4^{\prime }}\;+\;2V_{O}^{••}\;+\;\mathrm{Ce}O_{2}^{surf.}$$

where ${\mathrm{Ce}}_{\mathrm{Ce}}^{\times }$—cerium at a regular cerium lattice site; $O_{O}^{\times }$—oxygen at a regular oxygen lattice site; $V_{\mathrm{Ce}}^{4^{\prime }}$—negatively charged cerium vacancy; $V_{O}^{••}$—positively charged oxygen vacancy; $\mathrm{Ce}O_{2}^{surf.}$—CeO_2_ species at the surface.

According to Anti-Frenkel:

(13) $$O_{O}^{\times }↔V_{O}^{••}+{O_{i}}^{\prime \prime }$$

where $O_{O}^{\times }$—oxygen at a regular oxygen lattice site; $V_{O}^{••}$—positively charged oxygen vacancy; ${O_{i}}^{\prime \prime }$—negatively charged interstitial oxygen ion.

Therefore, while the hybrid mixed oxide support does not alter the reaction order, it significantly accelerates the reaction rate, as reflected by a reduction in the activation energy to as low as 10.7 kJ/mol for Pt^0.25^/CeO_2_^5^/TiO_2_, which is more than five times lower than the 60.3 kJ/mol observed for the non-catalytic reaction [14]. Moreover, the developed Pt^0.25^/CeO_2_^5^/TiO_2_ catalyst exhibits substantially higher catalytic activity than the related catalysts reported in previous studies and summarized in Table 4.

Despite differences in experimental conditions among the studies, the obtained results indicate the high efficiency of the developed catalyst for iron(II) oxidation under strongly acidic conditions at 25 °C.

## 4. Conclusions

The presence of iron(II) ions as impurities hinders the leaching and refining of magnesium ores. The most viable technological solution is to oxidise Fe^2+^ → Fe^3+^ and then precipitate Fe^3+^ as goethite. However, the slow kinetics of this process, particularly in highly acidic media, necessitate the development of new catalysts. One effective way of synthesising such catalysts is to use hybrid supports, where a finely dispersed cerium dioxide sol is deposited onto titanium dioxide powder. This increases the support’s specific surface area, ensures a uniform distribution of platinum particles, and enhances their durability in acidic environments due to the preferential interaction between the platinum particles and CeO_2_.

This study demonstrates that the catalyst Pt^0.25^/CeO_2_^5^/TiO_2_ is highly effective in oxidising Fe^2+^ in 0.3 M HNO_3_, outperforming samples supported on pure titanium dioxide or cerium dioxide. The rate constant is 0.304 L/(mol·min) at room temperature. Notably, the activation energy is just 10.7 kJ/mol and over five times lower than that of the non-catalysed process. This result was achieved by selectively depositing platinum particles onto highly dispersed cerium dioxide particles (average size ~5 nm), synthesised using the sol–gel method, and then depositing them onto titanium dioxide. Using a hybrid support ensures a high density of oxygen vacancies at the interface between the platinum (Pt) and mixed CeO_2_ and TiO_2_ phases. The formation of abundant adsorbed oxygen species enhances the catalytic activity of the Pt^0.25^/CeO_2_^5^/TiO_2_ catalyst. The Pt^0.25^/CeO_2_^5^/TiO_2_ catalyst exhibited high catalytic activity in strongly acidic media and retained approximately 75% of its initial activity after 15 consecutive cycles.

Catalysts of this type can be successfully used in the ore leaching process, including for magnesium ores, to effectively remove iron impurities in a strongly acidic environment. However, the catalyst composition proposed in this study requires further refinement and optimization.

## Figures and Tables

**Figure 1:**
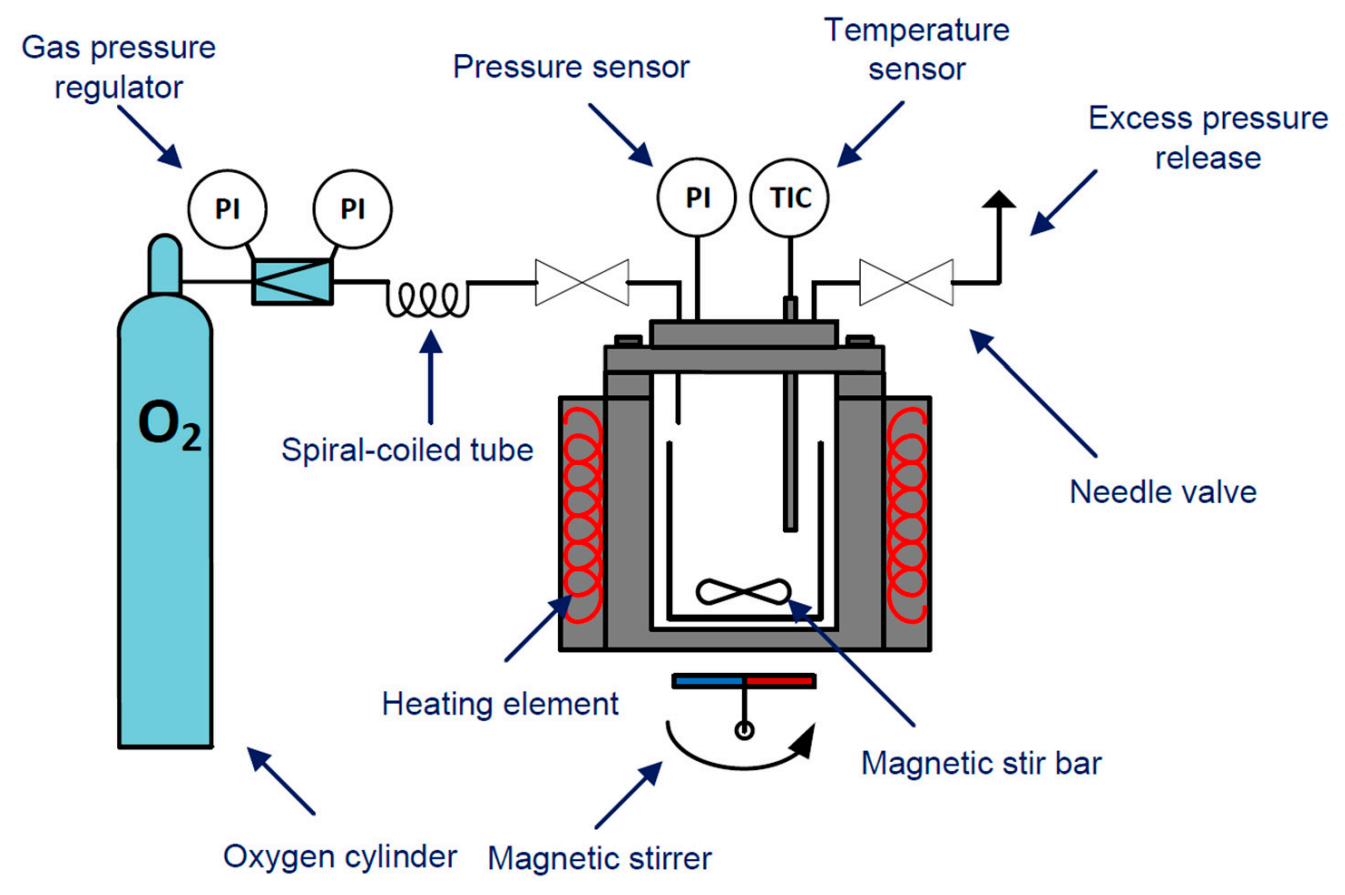
Schematic diagram of the experimental reactor setup for catalytic testing.

**Figure 2:**
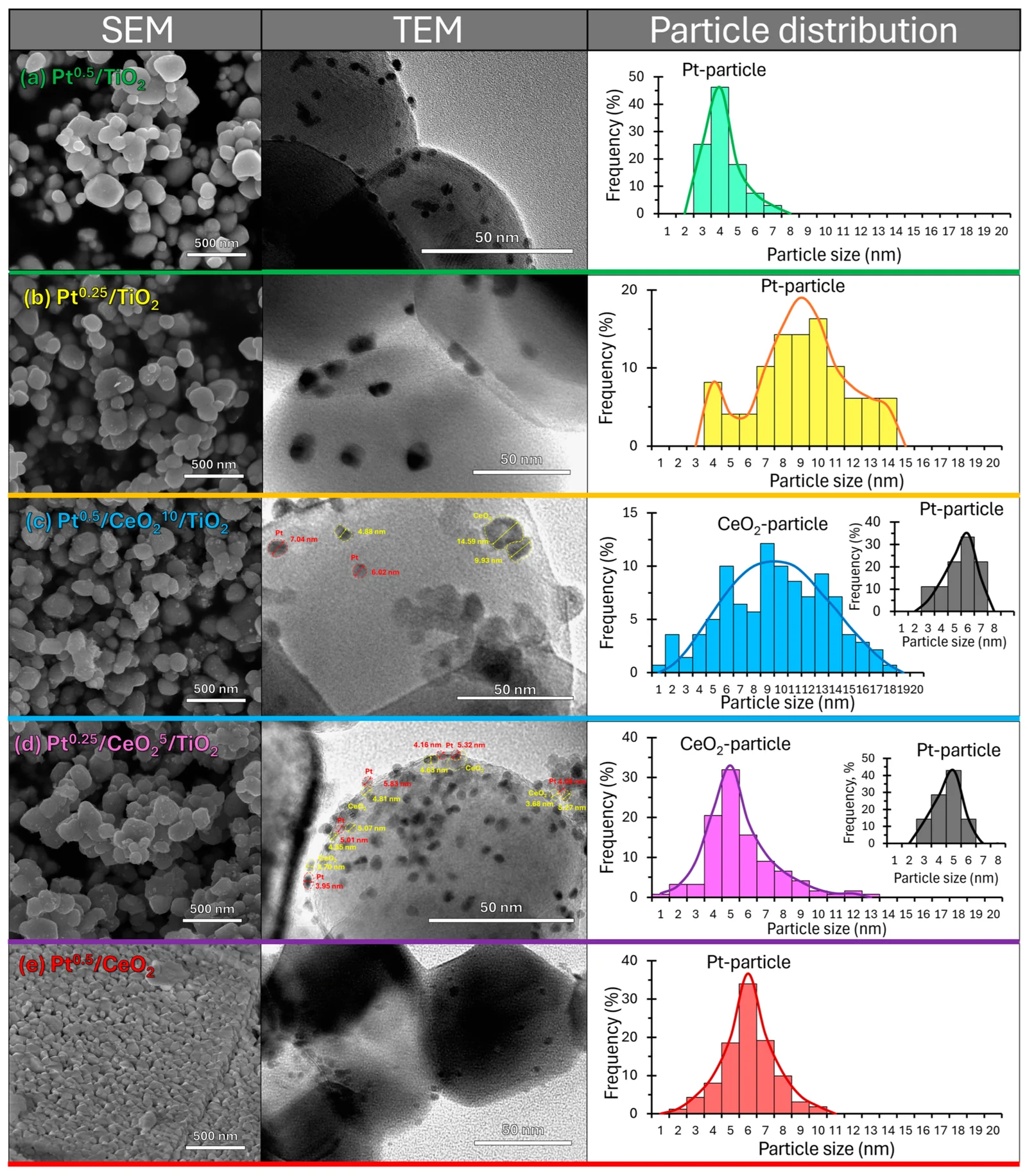
SEM and TEM images and particle size distributions of platinum and cerium oxide in the following catalysts: (**a**) Pt^0.5^/TiO_2_, (**b**) Pt^0.25^/TiO_2_, (**c**) Pt^0.5^/CeO_2_^10^/TiO_2_, (**d**) Pt^0.25^/CeO_2_^5^/TiO_2_, (**e**) Pt^0.5^/CeO_2_.

**Figure 3:**
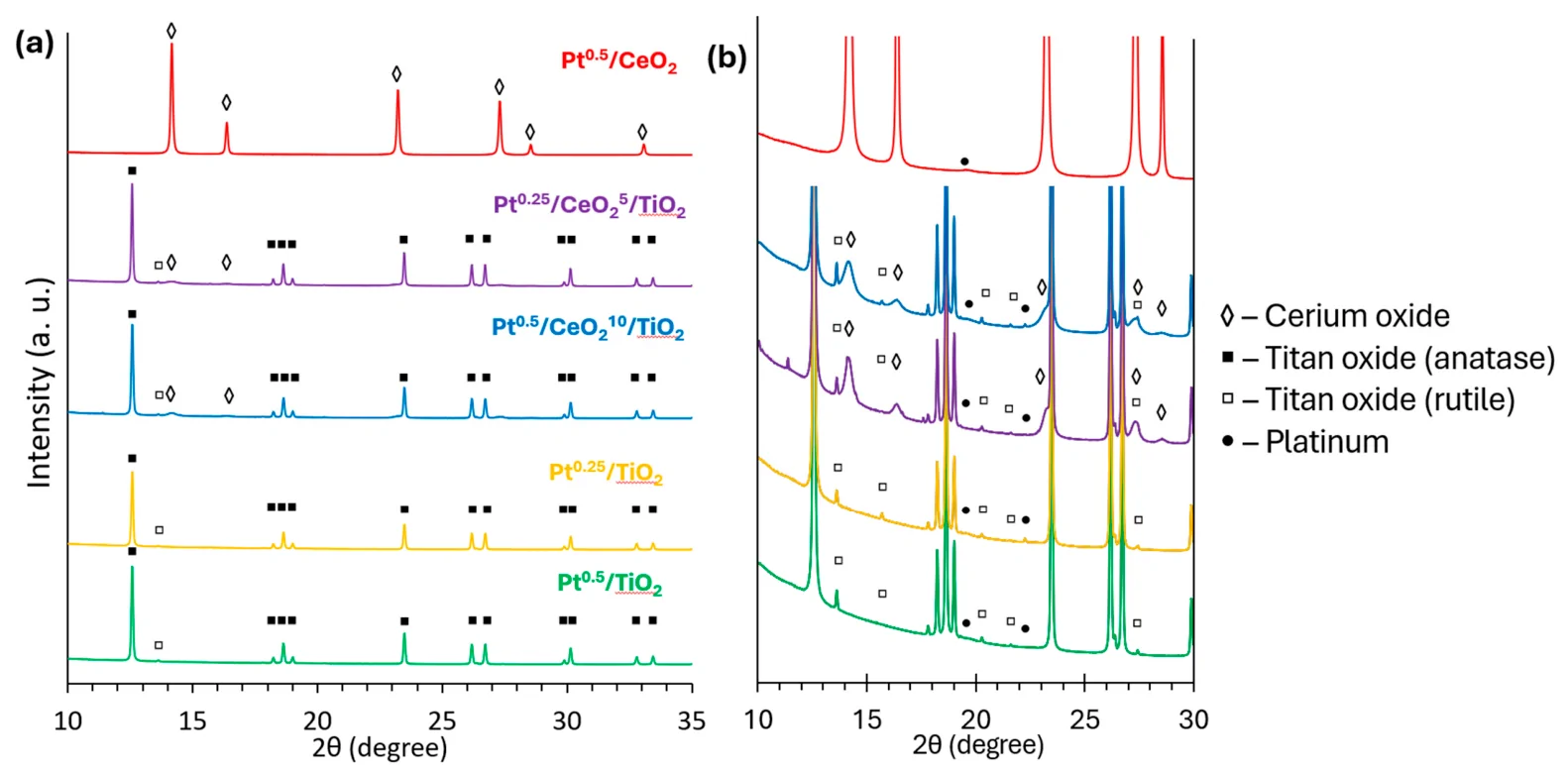
Powder XRD (**a**) of Pt^0.25^/TiO_2_, Pt^0.5^/TiO_2_, Pt^0.5^/CeO_2_^10^/TiO_2_, Pt^0.25^/CeO_2_^5^/TiO_2_, Pt^0.5^/CeO_2_ and (**b**) the enlarged view in the 2θ range of 10–30°.

**Figure 4:**
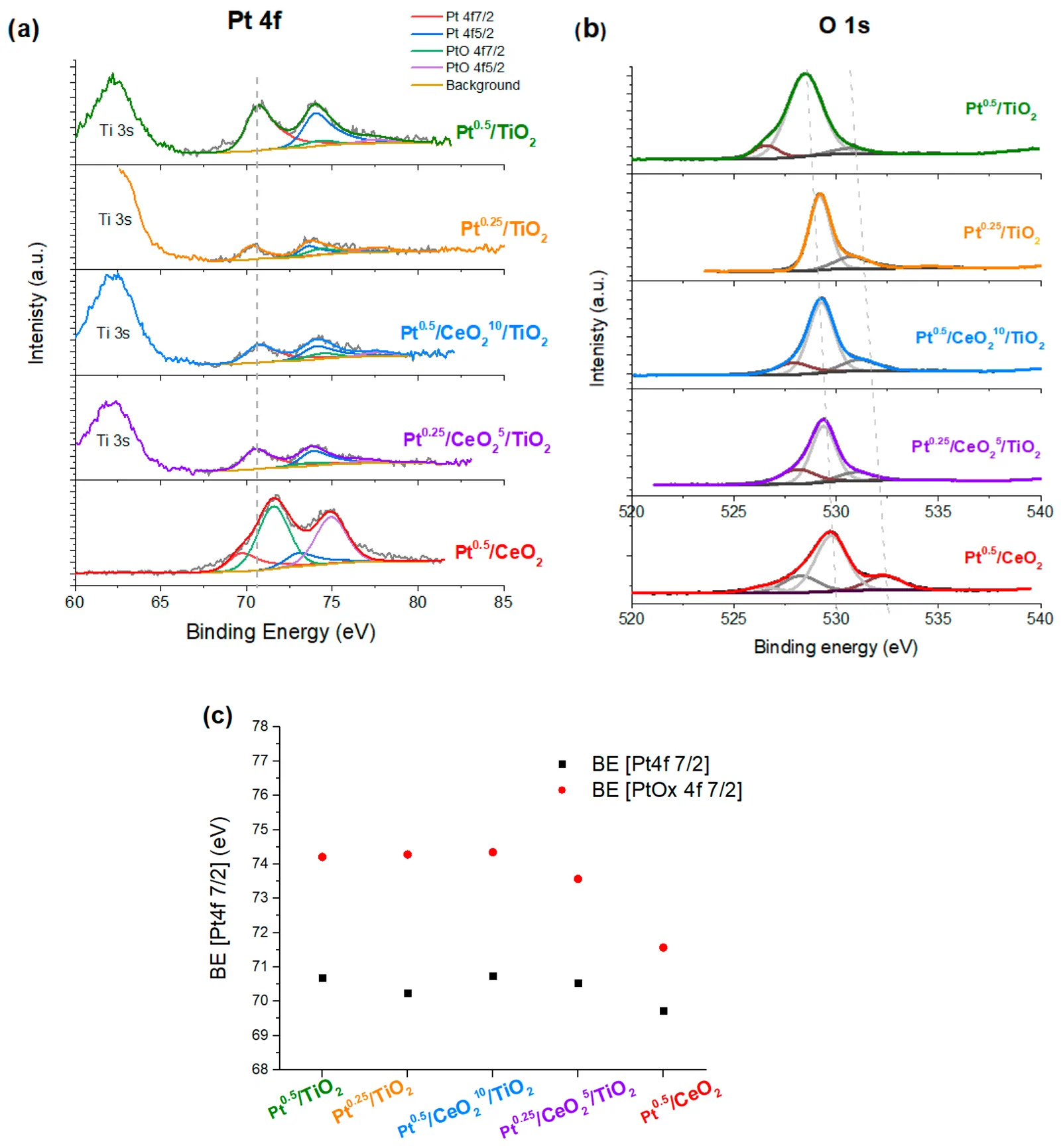
XPS spectra of various catalysts: Pt 4f (**a**), O 1s (**b**), and BE (**c**).

**Figure 5:**
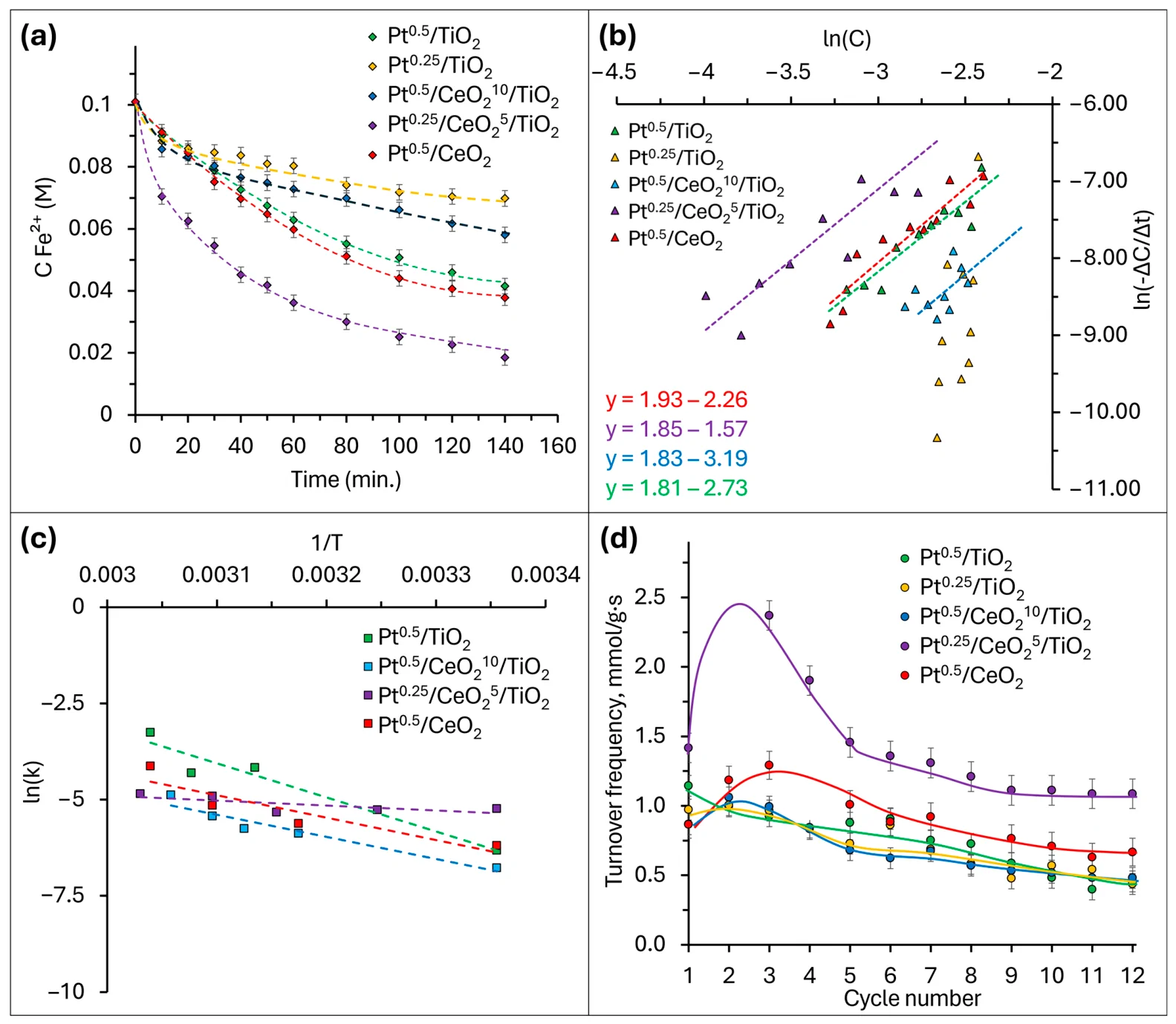
Kinetic data of various catalysts: (**a**) kinetic curves, (**b**) van Hoff’s plots, (**c**) Arrhenius plots, and (**d**) TOF curves.

**Figure 6:**
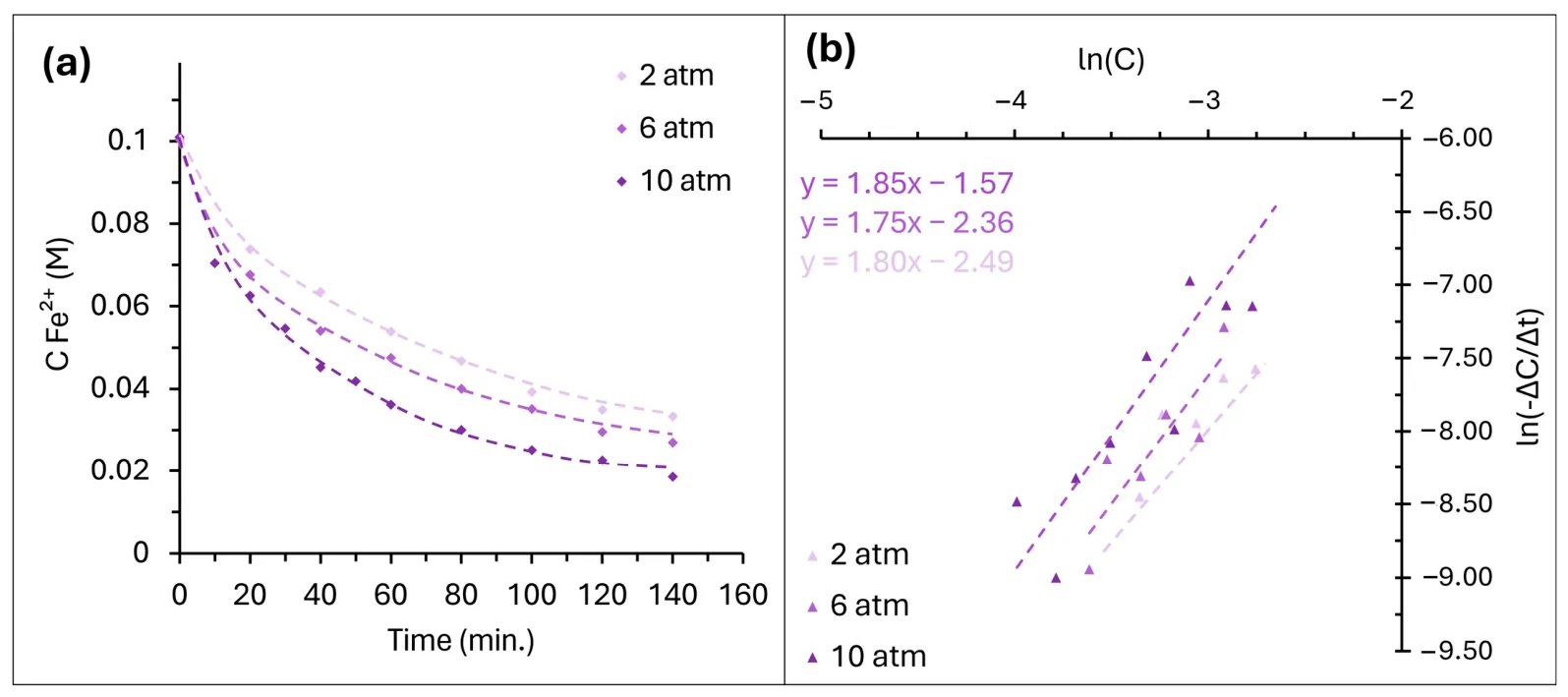
Effect of oxygen pressure on the kinetics of Fe^2+^ oxidation over the Pt^0.25^/CeO_2_^5^/TiO_2_ catalyst: (**a**) kinetic curves at different O_2_ pressures and (**b**) reaction order determination with respect to oxygen.

**Table 1:** Results of elemental analysis (XRF) and specific surface area (SSA) calculated by BET of catalysts.

No.	Sample	Elements Content, mas. %			SSA BET, m^2^/g
		Ti	Ce	Pt	
1	Pt^0.5^/TiO_2_	99.20 ± 9.92	–	0.80 ± 0.08	5.78
2	Pt^0.25^/TiO_2_	99.72 ± 9.97	–	0.28 ± 0.03	5.20
3	Pt^0.5^/CeO_2_^10^/TiO_2_	89.35 ± 9.94	10.15 ± 0.11	0.50 ± 0.05	7.40
4	Pt^0.25^/CeO_2_^5^/TiO_2_	93.65 ± 9.94	6.20 ± 0.62	0.15 ± 0.02	9.12
5	Pt^0.5^/CeO_2_	–	99.30 ± 9.93	0.70 ± 0.07	3.17

**Table 2:** The XPS data.

No.	Sample	Pt 4f 7/2, mass. %	Pt 4f 5/2, mass. %	PtO 4f 7/2, mass. %	PtO 4f 5/2, mass. %	O 1s ads, mass. %
1	Pt^0.5^/TiO_2_	49.86	37.4	7.28	5.46	5.85
2	Pt^0.25^/TiO_2_	32.72	24.54	24.42	18.32	3.76
3	Pt^0.5^/CeO_2_^10^/TiO_2_	42.11	31.58	15.04	11.27	15.56
4	Pt^0.25^/CeO_2_^5^/TiO_2_	47.46	35.6	9.68	7.26	13.52
5	Pt^0.5^/CeO_2_	11.38	8.54	45.76	34.32	16.52

**Table 3:** The kinetic data.

No.	Sample	Rate Constant (T = 25 °C), L·mol^−1^·min^−1^	Reaction Order	Activation Energy, kJ/mol
1	Pt^0.5^/TiO_2_	0.093	2	70.6
2	Pt^0.25^/TiO_2_	–	–	–
3	Pt^0.5^/CeO_2_^10^/TiO_2_	0.069	2	47.9
4	Pt^0.25^/CeO_2_^5^/TiO_2_	0.304	2	10.7
5	Pt^0.5^/CeO_2_	0.097	2	48.3

**Table 4:** Comparison of catalytic activities of different catalysts.

Catalysts	Rate Constant	Activation Energy, kJ/mol	Conditions	References
Cu^2+^	–	36.5	0.6 M H_2_SO_4_, C (CuSO_4_) = 4.0 × 10^−3^ M, 180 °C	[15]
Carbon	0.187 M^−0.5^·min^−1^	–	80 °C	[18]
Pt^0.25^/CeO_2_^5^/TiO_2_	0.304 L/mol^−1^·min^−1^	10.7	25 °C, 1.01 MPa	Present work
Without catalyst	–	62.1	1 M H_2_SO_4_, O_2_ 0.18 MPa	[14]

## Data Availability

The original contributions presented in this study are included in the article. Further inquiries can be directed to the corresponding author.

## Abbreviations

The following abbreviations are used in this manuscript:

SSA	Specific surface area
CSA	Cross-sectional area
TEM	Transmission electron microscopy
SEM	Scanning electron microscopy
BET	Brunauer–Emmett–Teller theory
TOF	Turnover frequency
